# Channelling actions in motor cortex: how *I*_h_ gates cortical control of movement

**DOI:** 10.1113/JP273363

**Published:** 2017-02-01

**Authors:** Julian J. Ammer, Ian Duguid

**Affiliations:** ^1^ Centre for Integrative Physiology and Patrick Wild CentreUniversity of EdinburghHugh Robson Building, George SquareEdinburghEH8 9XDUK

Whether we run, throw a ball or play the guitar, volitional movement requires the generation of descending motor commands to coordinate appropriate sequences of muscle contractions. For centuries researchers have been fascinated by the simple but fundamentally important question of how movement representations are organised in the brain. As early as 1870, Fritsch and Hitzig demonstrated that electrical stimulation of specific cortical areas in the dog elicited discrete, reproducible motor movements (Fritsch & Hitzig, 1870; for an English translation see Carlson & Devinsky, 2009). This initial discovery prompted a wealth of human and animal electrical stimulation studies that developed the idea of a structured somatotopic map of the external musculature in primary motor cortex (M1), now commonly referred to as the ‘cortical homunculus’. Since then, however, a more complex picture has evolved.

The development of high‐resolution intracortical microstimulation (HR‐ICMS) – a technique in which a single microelectrode is used to produce focal, layer‐specific cortical stimulation – revolutionised cortical mapping experiments (Asanuma and Sakata, 1967). With the application of HR‐ICMS it became increasingly apparent that describing M1 as a simple topographic map of individual muscles was an oversimplification. Instead, M1 contains representations of more complex, coordinated movements, where multiple different joint and muscle movements can be evoked from the same site depending on the strength and duration of the stimulus (Graziano, 2006). Although significant advances have been made in mapping movement representations in M1, the cellular and circuit mechanisms that control the selection and execution of simple *versus* complex motor movements remain elusive.

In a recent issue of *The Journal of Physiology*, Boychuk *et al*. (2017) explore the importance of the hyperpolarisation‐activated cation current (*I*_h_) in regulating single and multiple movement representations in M1. They begin by using HR‐ICMS to map sites in forelimb motor cortex where simple (i.e. digit, wrist, elbow or shoulder) or multi‐joint (i.e. uni‐ or bilateral combinations of digit, wrist, elbow and shoulder) movements can be elicited. By applying the selective hyperpolarisation‐activated, cyclic nucleotide‐gated (HCN) channel blocker ZD7288, they show an increase in the proportion of HR‐ICMS sites that elicit multi‐joint movements, without an increase in the overall area of the movement representation map. Thus, *I*_h_ appears to play a pivotal role in regulating forelimb movement complexity within localised areas of M1, rather than a more general role in controlling cortical excitability.

Boychuk *et al*. then explore whether experience can alter motor representations in M1 through changes in *I*_h_ expression levels. They show that electrically induced seizures reduce *I*_h_ currents in M1 layer 5 (L5) pyramidal neurons while increasing the proportion of sites eliciting multi‐joint movements. In contrast to ZD7288 application, seizure induction also enhances the overall area of the M1 movement representation map. However, given the widespread cellular and systems level changes occurring during seizure induction, it is not clear whether this effect results from the selective modulation of *I*_h_ or other off‐target effects. To address this potential caveat, the authors use a global HCN1 knock‐out mouse to demonstrate that *I*_h_ is critical for regulating both the threshold and the extent of ICMS‐driven multi‐joint movement representations in forelimb M1.

But do changes in *I*_h_ affect behaviourally relevant movements? Boychuk *et al*. show that HCN1 knock‐out mice display atypical reaching movements in a single pellet reaching task. The behavioural effects manifest as reaching component errors where digit to midline and elbow to midline movements are impaired. Since the global deletion of HCN1 could affect long‐range M1 inputs and/or activity in downstream motor areas, Boychuk *et al*. perform an elegant series of experiments using intracortical microinfusion of ZD7288 to selectively abolish *I*_h_ in forelimb M1. They show that task engagement is unaltered, i.e. rats perform a similar number of reaching attempts, but the accuracy with which the task is executed is significantly reduced. These results provide compelling evidence to suggest a critically important role for *I*_h_ in coordinating the execution of behaviourally relevant forelimb movements.

What are the implications for understanding cortical motor control? The findings clearly show that HCN channels play a previously unappreciated role in ‘gating’ single or multi‐joint movements and that *I*_h_‐mediated regulation of motor cortical excitability is a necessary prerequisite for performing accurate, skilled forelimb reaches. Given the predominant expression of *I*_h_ in M1 corticospinal neurons (Sheets *et al*. 2011) and hierarchical intralaminar L5 connectivity patterns that limit across‐cell‐class connections (Kiritani *et al*. 2012), HCN1 channel modulation could provide a cell type‐specific mechanism to differentially regulate corticospinal neuron recruitment depending on changing behavioural demands. Moreover, *I*_h_ is under potent noradrenergic neuromodulatory control, profoundly altering synaptic integration and output selectively in M1 corticospinal neurons (Sheets *et al*. 2011). It therefore invites speculation that long‐range noradrenergic input from the locus coeruleus and subsequent regulation of *I*_h_ in corticospinal neurons could provide a mechanism for channelling information to the spinal cord during movement selection and execution. Outstanding issues still to be addressed include which L5 cell type(s) underpin *I*_h_‐mediated changes in motor cortical output *in vivo*, whether apical and/or basal dendritic HCN channels regulate pyramidal neuron excitability during behaviour, and whether *I*_h_ shapes the precision and accuracy of other simple and complex motor behaviours.

In summary, Boychuk *et al*. demonstrate a crucial role for *I*_h_ in controlling single and multi‐joint movement representation in M1, providing exciting new insights into the possible cellular and circuit mechanisms that coordinate cortical control of movement.

## Additional information

### Competing interests

None declared.

### Author contributions

Both authors have approved the final version of the manuscript and agree to be accountable for all aspects of the work. All persons designated as authors qualify for authorship, and all those who qualify for authorship are listed.
